# HER3: Toward the Prognostic Significance, Therapeutic Potential, Current Challenges, and Future Therapeutics in Different Types of Cancer

**DOI:** 10.3390/cells12212517

**Published:** 2023-10-25

**Authors:** Avisek Majumder

**Affiliations:** Department of Medicine, University of California, San Francisco, CA 94158, USA; avisek.majumder@ucsf.edu

**Keywords:** targeted therapy, structure-function analyses, small-molecule inhibitors, monoclonal antibody, antibody–drug conjugate (ADC), epidermal growth factor receptor (EGFR), human epidermal growth factor receptor 2 (HER2), non-small cell lung cancers (NSCLC), breast cancer

## Abstract

Human epidermal growth factor receptor 3 (HER3) is the only family member of the EGRF/HER family of receptor tyrosine kinases that lacks an active kinase domain (KD), which makes it an obligate binding partner with other receptors for its oncogenic role. When HER3 is activated in a ligand-dependent (NRG1/HRG) or independent manner, it can bind to other receptors (the most potent binding partner is HER2) to regulate many biological functions (growth, survival, nutrient sensing, metabolic regulation, etc.) through the PI3K–AKT–mTOR pathway. HER3 has been found to promote tumorigenesis, tumor growth, and drug resistance in different cancer types, especially breast and non-small cell lung cancer. Given its ubiquitous expression across different solid tumors and role in oncogenesis and drug resistance, there has been a long effort to target HER3. As HER3 cannot be targeted through its KD with small-molecule kinase inhibitors via the conventional method, pharmaceutical companies have used various other approaches, including blocking either the ligand-binding domain or extracellular domain for dimerization with other receptors. The development of treatment options with anti-HER3 monoclonal antibodies, bispecific antibodies, and different combination therapies showed limited clinical efficiency for various reasons. Recent reports showed that the extracellular domain of HER3 is not required for its binding with other receptors, which raises doubt about the efforts and applicability of the development of the HER3-antibodies for treatment. Whereas HER3-directed antibody–drug conjugates showed potentiality for treatment, these drugs are still under clinical trial. The currently understood model for dimerization-induced signaling remains incomplete due to the absence of the crystal structure of HER3 signaling complexes, and many lines of evidence suggest that HER family signaling involves more than the interaction of two members. This review article will significantly expand our knowledge of HER3 signaling and shed light on developing a new generation of drugs that have fewer side effects than the current treatment regimen for these patients.

## 1. Introduction

**Figure 1:**
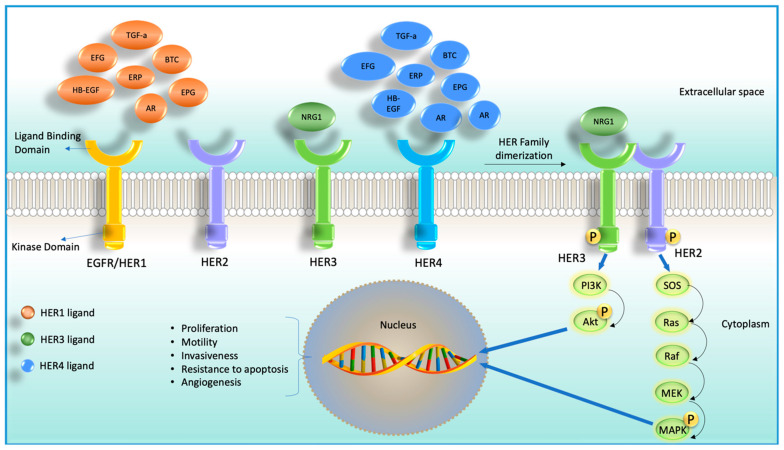
Cartoon diagram showing four receptor tyrosine kinases (RTKs) members and their corresponding ligands (HER2 does not have any ligand) on the left-hand side. On the right-hand side, it shows ligand-dependent heterodimerization of HER2 and HER3 and their downstream signaling cascade.

Although after 2020, the diagnosis and treatment of cancer were adversely affected due to the coronavirus disease 2019 (COVID-19) pandemic, in 2022, 1,918,030 new cancer cases and 609,360 cancer deaths are projected in the United States [1]. Despite the enormous efforts in advancing anti-cancer agents, it is still challenging to treat cancer due to the development of drug resistance. Accumulation of mutations in solid tumors imposes even more pharmacological challenges. The ability of tumor cells to survive, grow, migrate, and invade depends on the interaction of cell surface receptors and many growth factors [2]. Some of these growth factors were found to bind with cell surface localized receptor tyrosine kinases (RTKs), as shown in Figure 1. RTKs have been an attractive target for developing anti-cancer agents due to their accessibility and the ability to block the catalytic kinase function using small molecules [3,4,5]. There are four types of RTKs: human epidermal growth factor receptor (HER) 1 (EGFR, ErbB1), HER2 (Neu, ErbB2), HER3 (ErbB3), and HER4 (ErbB4). These RTKs are generally expressed in epithelial, mesenchymal, and neuronal tissues and are found to regulate cell division, proliferation, and differentiation [6,7]. Among these four members of RTKs, EGFR and HER2 are the most studied targeted molecules in cancer therapy [8]. 

Although HER3 has long been underestimated for targeting, recent studies found its emerging role in oncogenesis, tumor progression, and drug resistance [9]. HER3 is a unique family member of HER family proteins for many reasons. Unlike other HER family members, HER3 cannot form a homodimer, lacks/almost no intracellular kinase activity, and can form a heterodimer with other non-HER family proteins [10,11,12,13]. When HER3 is activated via binding with other receptors, it primarily activates PI3K/Akt signaling [14,15]. Additionally, HER3 was also reported to activate the MAPK cascade, Janus kinase (JAK), and proto-oncogene c-Src (SRC) signaling pathways, all of these pathways are tumorigenic [16,17]. It is now well recognized that HER3 can restore the signaling function via the PI3K/Akt axis under EGFR-targeted inhibition [18]. 

Even though it is now recognized that HER3 is a prime target for therapy, all the drugs available in the market are targeted against only two members of the HER/ERBB family proteins, namely EGFR and HER2. As it is impossible to target HER3 via conventional methods through inhibition of kinase activity, many pharmaceutical companies have been trying to develop different agents, including monoclonal antibodies and drug conjugates. Nonetheless, many drugs are developed targeting HER3 and are currently in various stages of clinical development. Interestingly, some studies also suggest that the extracellular domain of HER3 is not required for signaling, which raises an important question about the applicability of that specific therapy that targets the extracellular domain of HER3 [19]. 

It is suggested that signal generation in the HER3 occurs through an asymmetric kinase dimerization, where one kinase allosterically activates the other kinase [20]. Although it has been assumed that signaling and oncogenic functions of HER3 involve heterodimerization with another receptor, no crystal structure has been developed for a fully active HER3 signaling complex, so it is still known whether HER3 forms a dimer or there are more than two binding partners involved for this interaction. In fact, higher-order oligomerization is the widely believed mode of signaling in the HER family. The involvement of oligomeric assemblies in HER family signaling has been suggested by several studies involving fluorescent tracing and other techniques [21,22,23,24,25,26,27]. However, the interfaces involved in these interactions and their functional relevance remain to be defined. Hence, it is essential to critically review the 30 years of bench-to-bedside research to develop novel pharmacological interceptors of HER3. Such understanding can lay the foundation for a newer generation of drugs that can significantly enhance the efficacy of TKIs and prove highly effective without the need for cytotoxic agents.

## 2. HER3 Structure and Mechanism of Activation

**Figure 2:**
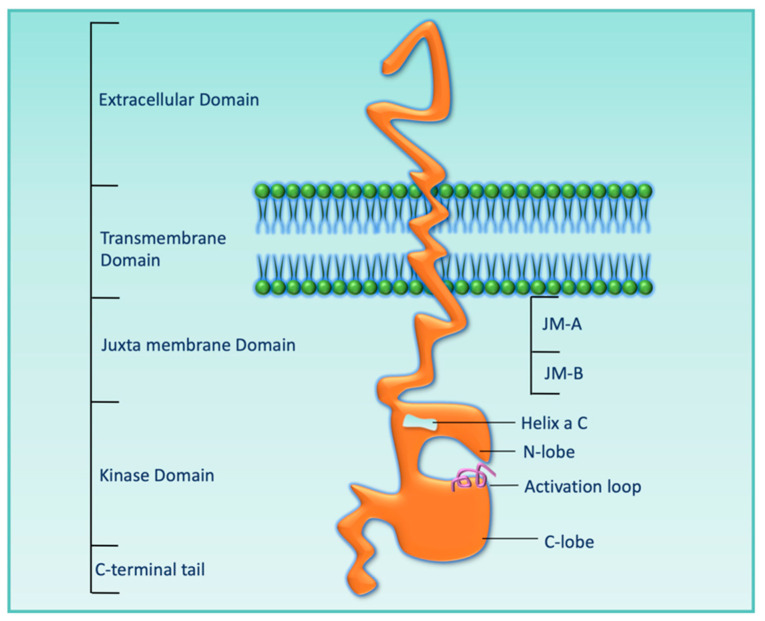
Cartoon diagram showing the extracellular domain, transmembrane domain, juxta membrane domain, kinase domain, and C-terminal tail of HER3.

HER3 was discovered by Kraus et al. in 1989, and it maps to chromosome 12q13 [28]. HER3 protein mainly has five domains: the extracellular domain, transmembrane domain, juxta membrane domain, kinase domain, and C-terminal tail, as shown in Figure 2. The extracellular domain consists of four subdomains, from domain I to domain IV. Upon ligand binding, a structural conformational change occurs that converts an untethered/inactive form to a tethered/active form, which enables it for heterodimerization with other HER receptors. Although the HER3 kinase domain (KD) shares 83% amino acid sequence identity with both EGFR and HER2, the presence of specific nonconservative amino acid residues (Cys-721, His-740, and Asn-815) in the HER3 KD makes its KD inactive [29]. 

Similar to other receptor tyrosine kinases (RTKs), the extracellular domain of HER3 exists in a reversible equilibrium between open (active) and closed (inactive) conformations. HER3 is active when this equilibrium shifts towards open conformation, allowing its dimerization arm within domain II to be exposed for dimerization (through the cysteine-rich CR1 region). So, when HER3 is in the open conform, it can dimerize with another HER family receptor. HER3 is the only family member of the HER family that does not form a homodimer. HER3 activation can occur in a ligand-dependent or ligand-independent manner. Conventionally, HER3 becomes activated via binding of its ligand NRG1 (also known as HRG) to the extracellular domain of HER3, which exposes its dimerization interface to be available for binding with other receptors. However, when any of its dimerization partners are present at sufficient concentration, it stabilizes HER3 in the open conformation transiently, known as ligand-independent activation. 

Signal generation in the HER family occurs through an asymmetric kinase dimerization, where one kinase allosterically activates the other kinase. In the case of the HER2-HER3 dimer, HER2 acts as a receiver kinase, phosphorylating tyrosine residues on the C-terminal tails of the activator kinases, i.e., HER3. After ligand binding, HER3 heterodimerizes with other HER family proteins that cross the phosphorylate C-terminal tail of HER3. This transphosphorylation at the HER3 C-terminal tail creates docking sites for p85, a regulatory subunit of protein phosphatidylinositol 3-kinase (PI3K) [30]. Studies reported that six tyrosine residues of the HER3 C-terminal tail are phosphorylated to recruit and activate PI3K [15,31]. Once activated, PI3K phosphorylates phosphatidylinositol bisphosphate (PIP2) and is converted to phosphatidylinositol trisphosphate (PIP3) in the plasma membrane [32]. PKB/Akt binds to PIP3, allowing PDK1 to phosphorylate the T308 residue of Akt to activate it [32]. The PI3K/Akt pathway also activates the mammalian target of rapamycin (mTOR) to control various biological processes, including survival, translation, nutrient sensing, cell cycle control, and metabolic regulation [32]. 

## 3. Expressions of HER3 in Different Types of Cancers

HER3 expression and activation levels go up during organogenesis in the postnatal maturation period [33,34]. Additionally, embryonic HER3 knockdown mice showed severely underdeveloped sympathetic ganglia and a partial lack of Schwann cells [35]; these suggest the importance of HER3 in the development of the fetal mouse brain. HER3 transcripts were reported in the human liver, kidney, and brain but not in heart or lung fibroblasts [28]. Like EGFR and HER2 expressions, HER3 expression was also reported in normal keratinocyte and glandular epithelium tissues; however, unlike EGFR and HER2, HER3 expression was not detected in fibroblasts, skeletal muscle, or lymphoid cells [28], suggesting the tissue-specific function of HER3 in ectodermal development. 

As physiological HER3 expression was reported in normal tissue, similarly, abnormal expression of HER3 has often been linked to a variety of different cancers, including breast cancer, colorectal carcinoma, squamous cell carcinoma of the head and neck, uveal melanoma, and gastric, ovarian, prostate, and bladder cancers, as shown in Table 1 [36,37,38,39]. Overexpression of HER3 protein has been linked to 50–70% of cases of breast cancer [40,41,42]. Upregulation of HER3 has been associated with metastasis, tumor volume, and risk of recurrence [43,44]. In colon cancer, HER3 overexpression has been associated with lymph node metastasis and poor progression [45,46,47,48]. Similarly, HER3 expression has been associated with increased metastasis and decreased overall survival in squamous cell carcinoma of the head and neck [49,50]. The above reports suggest the importance of HER3 in tumorigenesis and the need for targeting to inhibit tumor growth. 

Although HER3 is a membrane protein (like EGFR), it has also been reported to localize in the nucleus. A study reported in human breast cancer cells that HER3 predominantly localizes in the nucleus; however, upon ligand stimulation, it is transported to the cytoplasm [51]. Previous studies suggested that HER3 localization also has tissue-specific functions. In gastric carcinomas, HER3 nuclear expression is associated with vascular and lymphatic invasion [52]. Similarly, in prostate cancer, HER3 nuclear expression is correlated with tumor progression [53]. In contrast, nuclear HER3 expression is associated with favorable overall survival in uveal melanoma [54]. In contrast, membranous expression of HER3 is associated with decreased survival in head and neck squamous cell carcinoma [55].

**Table 1:** Expression of HER3 in different types of cancer detected by immunohistochemistry (IHC) in the selected studies.

Cancer Type	% of HER3 Overexpression	Antibody Used in IHC	Cutoff for Overexpression	Reference
Pancreatic	41.3%	Nanotools, Teningen, Germany	Moderate staining is observed in >10% of tumor cells (score 2+), and strong staining is observed in >10% of tumor cells (score 3)	[56]
Breast	43.0%	Clone 2F12, Labvision, Cheshire, UK	Positive: Optimal cutoffs for HER2:HER3 dimers were assessed by performing a minimum P value estimation using approximate 5% cutoffs across the entire dataset using relapse-free survival as an endpoint	[57]
	17.5%	IgG1, Neomarkers, UK	4-point scale, where 0 = no staining, 1 = light staining, 2 = moderate staining, and 3 = strong staining	[58]
Colorectal	17.0%	MAb-MS-725-P, Neomarkers, Fremont, CA	Membranous staining: >1% of tumor cells stained. Cytoplasmic staining: 2+: moderate immunostaining in >10% of tumor cells and 3+: strong immunostaining in >10% of tumor cells	[59]
	69.7%	Lab Vision, Fremont, CA Cytoplasmic and membrane Cytoplasmic	Cytoplasmic staining: 0: no staining or weak staining in <10% of tumor cells. membranous staining: 0: no staining in <10% of tumor cells; 1: weak staining in >10% tumor cells; 2+: moderate staining in > 10% tumor cells; 3: strong staining in >10% tumor cells	[59]
	20.9%	Clone C-17, 1:50; Santa Cruz, CA	Depending on the intensity of staining, HER3 expression was classified as weak, intermediate, or strong	[60]
Gastric	59.0%	Mouse monoclonal antibody, Neomarkers	2+ = moderate staining, and 3+ = strong staining	[61]
	34.0%	RB-9211 rabbit polyclonal, dilution 1:100, N terminal; Neomarkers, Fremont, CA	0 = <10% of positively stained cells; 1 = 10–25%; 2 = 26–50%; 3 = 51–75%; 4 = >75%	[52]
Melanoma	approximately 65.0%	Clone C-17, 1:50 dilution; SantaCruz, CA	Positive: high GIS > 6 (GIS: German immunohistochemical scoring)	[60]
Ovary	53.4%	C-17, rabbit polyclonal antibody; dilution: 1:25; Santa Cruz, CA	Positive: scores >8	[62]
Head and Neck	8.8%	RTJ.2, mouse monoclonal antibody; Santa Cruz, CA	Scores of 0, +1, +2, and +3 for increasing intensity	[55]
Cervix	55.5%	MS-725-P, mouse antibody; Neomarkers	Intensity scale 0–4 based on pixel density	[63]

## 4. Role of HER3 in the Genesis and Progression of Different Types of Cancer

As the KD of HER3 is inactive, it is an obligate binding partner with multiple HER family proteins to activate PI3K/Akt signaling [6]. PI3K/Akt signaling contributes to many biological functions (translation, survival, nutrient sensing, metabolic regulation, and cell cycle control) [64,65,66], which are also involved in tumorigenesis, suggesting the importance of HER3 in cancer development. This HER3/PI3 K/Akt signaling cascade has been linked to breast, ovarian, colon, gastric, and lung cancer [67]. 

Interestingly, a sequence analysis suggested the binding activity of HER3 cytoplasmic domains with not only PI3K but also other proteins such as GRB7, GRB2, SHC, and SRC [15]. GRB7 and GRB2 interact with HER3 through their SH2 domain, whereas SRC SHC interacts with HER3 through the PTB domain [67,68,69,70]. Studies found that GRB2 binds to HER3 only in the absence of GRB7 [69]. The SHC–HER3 interaction is essential for MAPK signaling [70]. 

Because HER3 function depends on binding with other receptors, HER3 cannot transform a normal cell into cancer cells [71,72]. The most favorable binding partner of HER3 is HER2. A study showed that introducing HER3 into NIH3T3 fibroblast cells results in a low level of colony growth, but when HER3 is transfected with HER2, it significantly induces colony growth compared to HER2 and HER3 alone [73]. Similarly, in vivo studies showed that the introduction of both EGFR and HER3 was not tumorigenic, whereas the introduction of HER2 and HER3 yielded significant tumorigenic growth compared to the combination of other HER family proteins. These suggest that the most potent binding partner of HER3 is HER2. 

HER3 is similarly important to its most potent binding partner, HER2 [38]. Studies also found that the knockdown of HER3 expression is more effective in inhibiting breast cancer cell growth than the knockdown of EGFR [38,74]. Interestingly, HER3 was found to preferentially dimerize with EGFR to induce cell proliferation, invasion, and migration in melanoma and pancreatic cancer [60,75]. Activation of HER3 via neuregulin-1 (NRG-1) was found to be enriched in a subset of SCCHN, and HER3 expression was associated with reduced survival in SCCHN [55,76]. Moreover, our previous studies in a panel of cancer cell lines of breast, bladder, colon, gastric, esophageal, lung, tongue, and endometrium cancer showed constitutive phosphorylation of HER3 in most of the HER2-amplified cancers [77]. Also, we noticed upon HER3 knockdown that overall tumor growth was substantially reduced in three HER2-amplified cancers from non-breast origin, suggesting HER3 has a vital role in tumorigenesis and tumor growth beyond breast cancer [77]. 

## 5. Role of HER3 Ligands in Tumor Growth and Resistance to Different Anti-Cancer Therapies

As discussed above, HER3, when activated via its ligand, dimerizes with another receptor (the most potent binding partner of HER3 is HER2) and transduces signals through the PI3K–AKT–mTOR pathway. Overexpression of HER3 ligands heregulin/neuregulin was shown to induce breast cancer progression. Studies also showed that resistance to TKIs may be due to compensatory upregulation of HER3 and that prerequisite for ligand-mediated activation of HER3. In breast cancer, it was found that high expression HER family receptor ligands play an essential role in developing resistance to anti-HER2 inhibitors [78,79,80,81]. Similarly, a study showed that the EGF ligand betacellulin and the HER3 ligand heregulin reduced the antiproliferative effect of anti-HER2/HER3 therapy [82]. The HER3 ligand neuregulin β1 was found to induce resistance to T-DM1 (a combination of Herceptin and the chemotherapy medicine emtansine). This study showed that neuregulin β1-mediated T-DM1 resistance was reversed by pertuzumab [83]. This study also found that HER2 non-overexpressing breast cancer cells (MDA-MB-175VII) that release autocrine heregulin are also resistant to T-DM1 [83]. Similarly, another study showed that neuregulin1 was upregulated after treatment with a p110α-specific inhibitor (BYL719), and this treatment failed to reduce tumor growth [84]. Heregulin has also been reported to induce anchorage-independent growth of breast cancer cells more potently than EGF [85]. As heregulin/neuregulin1 induces lapatinib resistance in *HER2*-amplified breast and gastric cancer cells, HER3 inhibition via siRNA was found to reverse this effect [81,86]. Additionally, activation of HER2 signaling by increased heregulin production was noted to be associated with acquired resistance to cetuximab in colorectal cancer cells [87].

## 6. Role of HER3 in Resistance to Different Anti-Cancer Therapies

### 6.1. HER3 as a Mediator of Resistance to Targeted Therapies

Many pharmaceutical companies developed small-molecule inhibitors and monoclonal antibodies against EGFR and HER2 because of their pro-oncogenic function in different types of cancer [6,88]. A monoclonal antibody, cetuximab, has been approved for SCCHN in combination with radiation therapy for locally advanced disease and platinum-based chemotherapy as a standard first-line systemic therapy. A clinical trial (EXTREME trial) showed that cetuximab had reduced the risk of death by 20% compared to patients receiving chemotherapy alone, and the median survival rate increased to 10.1 months [89]. Small-molecule inhibitors against EGFR, such as erlotinib and gefitinib, have also been shown to be significantly effective against EGFR-mutant lung cancer [90,91]. Trastuzumab (Herceptin; Genentech), a humanized monoclonal antibody against HER2, has shown clinical success [92]. Additionally, a phase III clinical trial in gastric cancer (ToGA trial) showed that trastuzumab and chemotherapy significantly improve overall survival without affecting the quality of life [93]. 

Multiple mechanisms have been proposed to associate HER3-mediated with drug resistance, including upregulation of HER3 expression [94], higher production of NRG1 [95,96], and HER2 amplification [87,97]. Studies found that NRG1-induced HER3 activation induced resistance to BRAF V600E inhibitor vemurafenib in colon cancer [98,99]. In accordance with these findings, anti-HER3 antibodies restore sensitivity to vemurafenib in BRAF-V600E mutant colon cancer [99]. Similarly, a study showed that triple blockade of HER2/HER3 signaling using trastuzumab, pertuzumab, and patritumab could overcome resistance to trastuzumab therapy in heregulin-expressing and HER2-positive breast cancer [100]. 

A previous report showed that HER3 plays a crucial role in HER2-mediated resistance to tamoxifen, and inhibition of HER3 expression reverses this resistance [101]. Similarly, HER3 expression and activation were also reported to induce resistance to fulvestrant [102]. Various studies noted that HER3 is a major player in trastuzumab and lapatinib resistance through the PI3K/AKT and Src signaling pathways [16,95,103,104]. HER3 was even reported to be linked to resistance to adjuvant chemotherapy in triple-negative breast cancer (TNBC) [105]. Likewise, a study showed that HER3 overexpression caused paclitaxel resistance in HER2-overexpressing BC cell lines [106]. HER3-mediated signaling was also found to be involved in inducing resistance in several other EGFR-mediated targeted therapies, including gefitinib and cetuximab [87,107]. Also, in lung cancer, MET amplification is reported to be involved in gefitinib and erlotinib resistance through upregulation of the HER3/PI3K signaling axis [12]. Similarly, upregulation of NRG1 and HER3 expression was found to induce resistance to anaplastic lymphoma kinase (ALK) and BRAF inhibitors in melanoma and thyroid cancer [94,99,108].

There are multiple antibodies targeting EGFR that have been approved for clinical use. However, patients developed resistance over time. Drug-induced compensatory upregulation of HER3 and sustained PI3 K/Akt activation have been reported to play an essential role in resistance to HER2-targeted therapy in breast cancer [18,30]. Similarly, in our previous studies in a panel of cancer cell lines of eight different types of cancer, we noticed that after treatment with lapatinib (an inhibitor of EGFR and HER2), almost all HER2-amplified cancers show only a transient HER3 inactivation and HER3 is eventually re-phosphorylated over time [77]. Studies also showed that prolonged exposure to EGFR inhibitors gefitinib, erlotinib, or the HER2 inhibitor AG-825 caused upregulation of HER3 and Akt phosphorylation [12,18]. This increased membrane expression of HER3 under prolonged treatment of HER TKIs regulated through Akt-mediated negative-feedback signaling [18]. After prolonged treatment with HER2 antibody, trastuzumab showed upregulation of EGFR and HER3 expression in breast cancer [109]. Another study showed that the upregulation of HER3 ligand heregulin is also a possible mechanism of cetuximab resistance in colorectal cancer patients [87]. Additionally, amplification of proto-oncogene MET causes gefitinib resistance by driving HER3-dependent activation of PI3K [12]. 

HER3 and HER2 expression was significantly correlated with gefitinib resistance but not cetuximab in non-small cell lung cancer and head and neck squamous cell carcinoma (HNSCC) [107]. A combination treatment of gefitinib and the HER2-HER3 dimerization inhibitor pertuzumab showed more effective growth inhibition than gefitinib alone on gefitinib-resistant HNSCC cell lines [107]. As in pancreatic cancer, HER3 dimerized with EGFR and siRNA-mediated inhibition of HER3 expression showed acquired resistance to erlotinib, suggesting that lack of HER3 expression makes these cells less dependent on EGFR [75]. This result suggests that this resistance-promoting function of HER3 may be caused by activated HER3, not the total HER3 expression. 

### 6.2. HER3 as a Mediator of Resistance to Hormonal Therapy, Chemotherapy, and Radiotherapy

HER3 is not only connected to resistance to targeted therapy; many studies also found HER3-mediated resistance to hormonal therapy, chemotherapy, and radiotherapy. One study found the critical role of HER3 in the progression of breast cancer cells, whereas inhibition of HER3 expression reduces resistance to reversed anti-estrogen receptor (ER) tamoxifen resistance in breast cancer cell lines [101]. Additionally, co-expression of HER2 and HER3 was reported to develop resistance to tamoxifen in breast cancer patients [110,111]. Similarly, elevated signaling via EGFR, HER2, and HER3 was found to induce resistance to ER agonist fulvestrant [112]. As a mechanism, another study showed that fulvestrant treatment enhances the HER3 expression as well as activation in an NRG1-dependent manner in breast cancer cells [102]. In triple-negative breast cancer, patients with high expression of both HER3 and EGFR were found to have worse 10-year survival after adjuvant chemotherapy compared to patients without adjuvant chemotherapy [105]. In castration-resistant prostate cancer (CRPC), NRG1 released from stromal cells induced antiandrogen resistance [113]. 

HER2 and HER3-mediated PI3K/AKT signaling was also reported to increase resistance for several chemotherapeutic agents, including 5-fluorouracil, paclitaxel, camptothecin, and etoposide in breast cancer cells [114]. Enhanced HER3 expression was also reported to cause paclitaxel resistance in erbB2-overexpressing breast cancer cells via the upregulation of Survivin [106]. A study found that a DNA-damaging agent, doxorubicin-induced HER3-PI3K-AKT signaling in ovarian cancer cells, and a combination of HER3 inhibition and doxorubicin reported increased apoptosis in the chemoresistant cells [115]. 

Moreover, using both in vitro and in vivo models, some studies also reported that ionizing radiation (IR) induces activation of EGFR, HER2, HER3, and HER4, and interestingly, silencing of HER3 inhibits the viability of cancer cells after treatment with IR [116,117]. 

## 7. Targeting HER3 and Its Challenges

HER family members are found to be expressed in endothelial and cardiac cells and play an essential role in proliferation and differentiation [118,119]. Similarly, HER3 KO was reported to exhibit ED13.5 lethality due to defective valve formation, pronounced heart defects, vasculature abnormalities, and specifically hypoplastic cardiac cushions with decreased mesenchyme [120,121]. In contrast to other HER family members, HER3 is also noted to be expressed in endocardial cushion cells and mesenchymal cells undergoing EMT [119]. HER3 is essential for developing and maintaining normal tissue and is intensely involved in developing a wide range of cancers [32]. HER3 has been shown to be involved in intrinsic or developed resistance against HER-targeting agents, and there is a considerable focus on optimizing this therapy [18,122]. Although EGFR and HER2 are widely known targets for therapy in cancer, HER3 has long been underestimated for cancer therapy. HER3 is the only family member of the HER family that lacks an active kinase domain, making it an obligate binding partner with other receptors for function. Studies showed that EGFR and HER2 are the most preferred dimerization partners of HER3 and also the most active heterodimers [74,123,124,125]. In view of the aberrant expression of HER3 and/or activation of HER3 and its ligand, NRG1 is associated with tumor progression and acquired resistance to EGFR and HER2-targeted therapies, and HER3 emerges as an interesting target [126,127]. As the HER3 kinase domain binds ATP with a Kd of approximately 1.1 μM and showed very little catalysis of autophosphorylation, it would not be beneficial to make small-molecule inhibitors against this KD [128]. Different pharmaceutical companies have been trying to target HER3 with a wide variety of approaches. 

Although HER3 knockout mice do not survive, inhibition of HER3 expression was found to be broadly safe in the clinic, with a low grade of side effects [121,129]. The pharmaceutical industry has been trying to develop antibodies against HER3, but they have shown limited clinical efficiency for several reasons. Some developed antibodies (e.g., seribantumab) block the NRG1-binding region. However, they do not consider the ligand-independent heterodimer formation [130]. Similarly, some antibodies (e.g., elgemtumab) were developed to target the closed conformation of HER3, but they do not address the conformation-dependent binding [131]. 

## 8. Development of HER3-Directed Therapy and Its Clinical Stages

Monoclonal antibodies (MoAbs) and antibody–drug conjugates have been widely used in targeting EGFR and HER2 in different cancers. Although HER3 has been ignored for a long time for its pseudo-KD, recently, HER3 came into the limelight due to its emerging role in tumor progression and drug resistance in various types of cancers. 

### 8.1. HER3-Targeting Monoclonal Antibodies and Clinical Trials

Pharmaceutical companies have been developing HER3 inhibitors and pan-ErbB inhibitors, which target HER3 as well as other HER family proteins. Many of these drugs are in the early clinical stages. As the HER3 KD is inactive, most of the TKIs cannot inhibit the ATP binding site of HER3. This challenge makes researchers develop antibodies that target the extracellular domain of HER3. Although several HER3-targeting monoclonal antibodies have been developed that showed limited toxicity profiles in clinical trials, objective responses were rarely observed (Table 2). Only three antibodies moved to phase II to III clinical trials: which include (I) Patritumab (U3-1287), a fully human HER3-directed monoclonal antibody that binds to the extracellular domain of HER3 and promotes receptor internalization [132]; (II) Seribantumab (MM-121), a fully human immunoglobulin G2 that binds to the ligand-binding domain of HER3 and inhibits HRG-mediated downstream PI3K/AKT signaling [133]; (III) Lumretuzumab (RO5479599), an immunoconjugate containing a humanized HER3-directed monoclonal antibody that binds to HER3 extracellular domain, inhibiting HER3 dimerization and EGFR-dependent signaling, and activates the immune system to exert antibody-dependent cellular cytotoxicity [134].

MM-121 is one of the fully humanized HER3 monoclonal antibodies that block the binding of HRG1 (a neuregulin-1 type I polypeptide) to HER3 [130]. MM-121 was found to inhibit tumor growth in pancreatic ductal adenocarcinoma (PDAC) through the Akt axis when combined with erlotinib [135]. Similarly, MM-121 was also found to induce sensitivity to gefitinib (an EGFR inhibitor) in gefitinib-resistant lung cancer cell lines [136]. In the lung cancer model, MM-121 showed an additive effect to reduce tumor growth when combined with the anti-EGFR antibody cetuximab [136]. In comparison, MM-111 is a bispecific antibody that binds both HER2 and HER3, shown to inhibit PI3K signaling. U3-1287 (AMG888) is the first fully humanized HER3 monoclonal antibody that induces rapid internalization of HER3 [137]. U3-1287 was reported to inhibit the growth of breast, lung, and colorectal cancer cell lines and inhibit tumor growth in pancreatic, NSCLC, and colorectal cancer xenograft models [138]. Similarly, pharmaceutical companies developed many antibodies targeting HER2 HER3 dimerization interfaces. Pertuzumab is one of these antibodies that inhibit HER2 and HER3 binding, and many clinical trials showed a significant benefit for HER2-positive breast cancer patients [68,139]. There have been many clinical trials with pertuzumab in different other cancer types as well. In addition to the above antibodies, many multitarget inhibitors (such as MEHD7945A, MP-470, and AZD8931) are still under clinical development.

**Table 2:** Development of HER3-directed monoclonal antibodies.

Monoclonal Antibodies	Study Population	Clinical Trial Phase	Adverse Events	Status Finding	Reference
Lumretuzumab	Advanced or metastatic NSCLC	NCT02204345 phase I + II	Gastrointestinal, hematological, and nervous system toxicities, but generally mild and manageable	Terminated Efficacy of lumretuzumab + carboplatin + paclitaxel is similar to chemotherapy alone	[140]
	Metastatic BC expressing HER3 and HER2	NCT01918254 phase I	Diarrhea and hypokalemia	Completed Lumretuzumab + pertuzumab + paclitaxel was correlated with a serious incidence of diarrhea	[141]
	Metastatic and/or locally advanced malignant HER3 + solid tumors of epithelial cell origin	NCT01482377 phase I	Gastrointestinal and skin toxicities	Completed Moderate clinical activity with toxicity manageable	[142,143]
ISU104	Advanced solid tumors	NCT03552406 phase I Dose escalation study (PART I) Dose-expansion study (PART II)	PART I: oral mucositis, pruritus, diarrhea, and fatigue PART II: anorexia, mucositis oral and diarrhea in monotherapy and diarrhea and acneiform rash in combination with cetuximab	Status unknown ISU104 was well tolerated up to 20 mg/kg/day without DLT and showed a disease control rate of 60% ISU104 monotherapy or with cetuximab was safe with promising clinical outcomes in recurrent or metastatic HNSCC treated with the combination	[144]
CDX-3379	Advanced cancer	NCT02014909 phase I	Diarrhea, fatigue, nausea, and rash	Completed CDX-3379 can be combined safely with cetuximab, erlotinib, vemurafenib, or trastuzumab at 15 to 20 mg/kg	[145]
	HNSCC	NCT02473731, phase I	Diarrhea, fatigue, and acneiform dermatitis, but mild or moderate	Completed CDX-3379 was well tolerated and associated with tumor regression	[146]
	Advanced stage NRAS mutant and BRAF/NRAS wild-type melanoma	NCT03580382 phase I + II		Terminated	
	Advanced HNSCC	NCT03254927 phase II		Terminated CDX-3379 in combination with cetuximab is well tolerated with signs of antitumor activity	[147]
	Thyroid cancer	NCT02456701 phase I		Completed Vemurafenib + CDX-3379 is safe and enhances efficacy for RAI uptake	[148]
AV-203	Metastatic or advanced solid tumors	NCT01603979 phase I		Completed AV-203 was well tolerated. RP2D is 20 mg/kg IV every 2 weeks. The PR in a patient with squamous NSCLC guarantees future testing of AV-203 in this indication	[149]
GSK2849330	Advanced HER3 + solid tumors	NCT01966445 phase I	Drug tolerated with no major issues	Completed GSK2849330 has a durable response in an exceptional responder with an advanced CD74–NRG1-rearranged IMA	[150]
	Advanced HER3 + solid tumors	NCT02345174 phase I	Decreased appetite and diarrhea	Completed Despite the restricted number of patients, an exploratory ID50 of 2 mg/kg and ID90 of 18 mg/kg have been reported	[151]
Seribantumab	Advanced NSCLC	NCT00994123 phase I + II	Diarrhea, rash, decreased appetite, fatigue, and nausea	Completed Phase I: no maximum tolerated dose was determined and the AE profile was similar between comparative treatment Phase II: there was no significant difference in PFS between monotherapy and combination therapy. However, retrospective analyses suggest that detectable NRG mRNA levels identified patients who may benefit from MM-121	[152]
	NSCLC expressing NRG	NCT02387216 phase II	Diarrhea, fatigue, and neutropenia in the combination treatment	Terminated Seribantumab does not improve PFS when added to docetaxel	[153]
	CRC, HNSCC, NSCLC, TNBC, and other tumors with EGFR dependence	NCT01451632 phase I	Part 1: fatigue, dermatitis acneiform, hypomagnesemia, diarrhea, decreased appetite, and hypokalemia Part 2: diarrhea, hypokalemia, nausea, fatigue, hypomagnesemia, decreased appetite, dermatitis acneiform, mucosal inflammation, dehydration, and weight decrease	Completed Unlike doublet treatment, seribantumab + cetuximab + irinotecan was difficult to tolerate. However, MM121 + cetuximab with and without irinotecan had no activity in the vast majority of patients with prior exposure to EGFR-directed therapy	[154]
	Advanced gynecologic and breast cancers	NCT01209195 phase I		Completed	
	ER +, HER2- BC, and TNBC	NCT01421472 phase II		Completed	
	Platinum-resistant or refractory recurrent/advanced ovarian cancers	NCT01447706, phase II	Diarrhea, vomiting, stomatitis, and mucosal inflammation	Completed	[155]
	Locally advanced or metastatic ER + and/or PR + and HER2- BC	NCT01151046 phase II	Diarrhea, nausea, fatigue, and arthralgia	Completed The addition of MM-121 to exemestane did not significantly prolong PFS in the unselected population	[156]
	CRC, NSCLC, and HNSCC	NCT02538627 phase I		Terminated	
	Advanced solid tumors	NCT00734305 phase I		Completed	
	Advanced solid	NCT01447225	Diarrhea, nausea, and fatigue,	Completed MM-121 can be administrated with	[157]
	Tumors	phase I	Anemia, vomiting, hypokalemia, decreased appetite, thrombocytopenia, peripheral edema, neutropenia, and constipation	Gemcitabine, pemetrexed, cabazitaxel, and carboplatin	
	Postmenopausal women with metastatic BC	NCT03241810 phase II		Terminated	
	Locally advanced or metastatic solid tumors	NCT01436565 phase I		Completed	
	NRG1 gene fusion-positive advanced solid tumors	NCT04383210 phase II		Active, not recruiting	
	An NRG1 fusion-positive metastatic pancreatic cancer patient	NCT04790695 phase II		Completed	
Patritumab	Advanced, refractory solid tumors	NCT01957280 phase I	The most frequently reported treatment-related AEs were gastrointestinal	Completed Well tolerated with no anti-patritumab neutralizing antibodies formation and with normal bioavailability	[158]
	EGFR wild-type subjects with locally advanced or metastatic NSCLC who have progressed on at least one prior systemic therapy	NCT02134015 phase III	In placebo + erlotinib, the most frequent AEs were rash, diarrhea, and fatigue, in patritumab + erlotinib were diarrhea, rash, and decreased appetite	Terminated Patritumab + erlotinib apparently do not have better results than placebo + erlotinib	
	Recurrent or metastatic HNSCC	NCT02633800 phase II	Rash, anemia, neutropenia, hypomagnesemia, and nausea	Terminated Patritumab + cetuximab + platinum was safe but not more efficacious than cetuximab + platinum	[159]
	EGFR treatment naïve subjects with advanced NSCLC who have progressed on at least one prior chemotherapy	NCT01211483 phase I + II	AE grade > 3 included diarrhea and rash	Completed Patritumab improved PFS in the NRG high, but not in the ITT population	
	Recurrent or metastatic HNSCC	NCT02350712 phase I	Skin and subcutaneous tissue disorders	Completed Patritumab (18 mg/kg loading dose, 9 mg/kg maintenance dose) with cetuximab, and platinum therapy was tolerated and active in HNSCC	[160]
	Advanced solid tumors	NCT01479023, phase I	Diarrhea, dizziness, fatigue, headache, hypertension, and weight loss	Terminated [64Cu]DOTA-patritumab and unlabeled patritumab are safe and well tolerated	[161]
	Newly diagnosed HER2 + metastatic BC	NCT01512199 phase I + II		Terminated	
	Advanced solid tumors	NCT00730470 phase I	Fatigue, diarrhea, nausea, decreased appetite, and dysgeusia	Completed Patritumab treatment was well tolerated and some evidence of disease stabilization was observed	[132]
Elgemtumab (LJM716)	Platinum-pretreated recurrent/metastatic HNSCC	NCT02143622 phase I + II		Withdrawn	
	Advanced HER2 + BC or gastric cancer	NCT01602406 phase I	Diarrhea, nausea, fatigue, and chills	Completed As of 4 October 2013, LJM716 demonstrated clinical activity in combination with trastuzumab in trastuzumab-resistant patients with an acceptable safety profile	[162]
	Metastatic HER2 + BC	NCT02167854 phase I	Diarrhea, hyperglycemia, hypokalemia, mucositis, and transaminitis	Completed The combination treatment of LJM716, BYL719 (PI3K inhibitor) and trastuzumab has antitumor activity in these pretreated HER2 + metastatic BC with PIK3CA mutations	[163]
	Patients with previously treated ESCC	NCT01822613 phase I + II		Completed	
	HER2 + BC, HER2 + gastric cancer, HNSCC and ESCC	NCT01598077 phase I	Diarrhea, decreased appetite, pyrexia, fatigue, nausea, infusion-related reactions, vomiting, constipation and dyspnea and anemia and hypomagnesemia	Completed LJM716 was well tolerated, with a manageable safety profile	[164]
	Japanese patients with advanced solid tumors	NCT01911936 phase I	Diarrhea, stomatitis, fatigue, pyrexia and paronychia	Completed LJM716 was well tolerated and a degree of tumor shrinkage was reported	[165]
REGN1400	Patients with advanced NSCLC, CRC, or HNSCC who progressed on prior erlotinib or cetuximab	NCT01727869 phase I	Rash, diarrhea, nausea, and hypomagnesemia	Completed REGN1400 as monotherapy or combined with erlotinib or cetuximab was generally tolerated	[166]
Sym013	Patients with advanced epithelial malignancies	NCT02906670 phase I + II		Terminated	

NSCLC: non-small cell lung cancer, BC: breast cancer, HNSCC: head and neck squamous cell carcinoma; AEs: adverse events; PFS: progression-free survival; ITT: intention to treat; ESCC: esophageal squamous cell carcinoma; CRC: colorectal cancer.

#### 8.1.1. HER3-Directed Monoclonal Antibodies in Breast Cancer

A phase 1 trial with sarilumab, paclitaxel, and trastuzumab in pretreated HER2-positive metastatic breast cancer confirmed manageable toxicities and encouraged preliminary activity [167]. A phase Ib trial, when lumretuzumab was added with pertuzumab and paclitaxel in 35 patients with HER3-positive HER2-low breast cancer, demonstrated a high incidence of diarrhea and a narrow therapeutic window [141]. In a biomarker analysis, a phase II study of seribantumab plus exemestane in patients with hormone receptor (HR)-positive HER2-negative metastatic breast cancer found a clinical benefit in the HRG-high subgroup [156]; however, this trial (SHERBOC trial) was prematurely terminated. 

#### 8.1.2. HER3-Directed Monoclonal Antibodies in NSCLC

In a phase II HERALD study, when patritumab was added to erlotinib, it did not prolong progression-free survival in 215 NSCLC patients and increased the risk of gastrointestinal toxicity [168]. At the same time, a phase III HER-3 lung trial did not confirm the efficacy of patritumab and erlotinib in the subgroup of EGFR wild-type NSCLC patients with high HRG expression [169]. Similarly, a randomized phase II trial of seribantumab in combination with erlotinib in patients with EGFR wild-NSCLC demonstrated progression-free survival benefits in patients with detectable HRG mRNA in the tumor [152]. Therefore, seribantumab received a fast-track designation for patients with HRG-positive NSCLC. 

#### 8.1.3. HER3-Directed Monoclonal Antibodies in Other Cancers

A phase II trial of an anti-HER3 monoclonal antibody, CDX-3379, and cetuximab in a population of heavily pretreated patients with head and neck squamous cell carcinoma (HNSCC) demonstrated antitumor activity with an acceptable safety profile [147]. Additionally, a first-in-human phase I study with ISU104 (ErbB3 monoclonal antibody) in patients with advanced solid tumors showed mucositis and diarrhea represented the most frequent treatment-related adverse events (TRAEs) [170]. 

### 8.2. HER3-Targeting Bispecific Antibodies and Clinical Trials

Blocking HER3 signals via monoclonal antibodies is challenging, so different bispecific antibodies have been developed for clinical tests, as summarized in Table 3. Among all these bispecific antibodies, the most promising one is the HER2/HER3-directed zenocutuzumab (MCLA-128), which is currently under a phase II clinical trial (NCT02912949). Zenocutuzumab was shown to reduce HRG-stimulated HER3 tumor growth and recruit natural killer (NK) cells into the tumor. Zenocutuzumab was evaluated in patients with NRG1 fusion-positive solid tumors, including NSCLC, breast cancer, and pancreatic cancer [171,172]. 

Another important bispecific antibody is duligotuzumab (MEHD7954A), which is an EGFR/HER3-directed bispecific monoclonal antibody. In a randomized phase II trial (MEHGAN study), duligotuzumab failed to improve clinical outcomes compared with cetuximab in squamous cell carcinoma of the head and neck (HNSCC) due to high incidence of gastrointestinal TRAEs [173]. In another phase II trial, the addition of duligotuzumab to FOLFIRI was not found to improve the clinical outcomes in patients with RAS exon 2/3 wild-type metastatic colorectal cancer compared with cetuximab plus FOLFIRI [174]. 

Moreover, the development of another bispecific HER3/IGF-1R-directed MoAb istiratumab (MM-141) was discontinued due to the negative results of the phase II CARRIE trial. This study showed that the addition of istiratumab to first-line nab-paclitaxel and gemcitabine did not show a clinical benefit in patients with metastatic pancreatic cancer with high IGF-1 serum levels [175].

**Table 3:** Development of HER3-directed bispecific antibodies.

Bispecific Antibodies	Study Population	Clinical Trial Phase	Adverse Events	Status Finding	Reference
Zenocutuzumab (Zeno, MCLA-128)	Solid tumors harboring an NRG1 fusion	NCT02912949 phase I + II	Infusion-related reactions, diarrhea, rash, and fatigue	Recruiting As of January 2017, MCLA-128 reported a safety profile and antitumor activity in pretreated metastatic breast cancer patients progressing on HER2 therapies	[176]
	A patient with advanced NRG1-fusion-positive solid tumor	NCT04100694 not given		Status and findings are not posted on www.clinicaltrials.gov (accessed on 20 October 2023)	
	Metastatic BC	NCT03321981 phase II	Neutropenia/neutrophil count decrease, diarrhea, asthenia/fatigue, and nausea	Active, not recruiting The combination of MCLA-128 + trastuzumab + vinorelbine is active in pretreated patients with HER2 + metastatic BC. The treatment is safe with manageable AEs	[177]
SI-B001	Locally advanced or metastatic epithelial tumors	NCT04603287 phase I		Recruiting	
	Recurrent and metastatic HNSCC	NCT05054439 phase II		Recruiting	
	Recurrent metastatic ESCC	NCT05022654 phase II		Recruiting	
	Recurrent and metastatic NSCLC	NCT05020769 phase II + III		Recruiting	
	EGFR/ALK wild-type recurrent or metastatic NSCLC	NCT05020457 phase II		Recruiting	
	Unresectable or metastatic digestive system malignancies (colorectal and gastric cancer)	NCT05039944 phase II		Terminated	
MM-111	Advanced, refractory HER2 amplified, NRG + BC	NCT01097460 phase I	Fatigue, diarrhea, and dyspnoea	Completed	
	Advanced, refractory HER2 amplified, NRG + cancers	NCT00911898 phase I		Completed	
	Advanced HER2 + solid tumors	NCT01304784 phase I	Anemia, acute renal failure (assessed as cisplatin-related), chest pain, decreased appetite, diarrhea, febrile neutropenia, hyperuricemia, hypokalemia, hyponatremia, hypophosphatemia, mucosal inflammation, nausea, neutropenia, stomatitis, thrombocytopenia, and vomiting	Completed Treatment with MM-111 and standard-of-care HER2-directed regimens was viable	[178]
	HER2 + carcinomas of the distal esophagus, gastroesophageal junction, and stomach	NCT01774851 phase II	Diarrhea, anemia, decreased appetite, alopecia, fatigue, nausea, vomiting, asthenia, neutropenia, constipation, and cough	Terminated MM-111 did not improve PFS or OS when added to paclitaxel + trastuzumab	[179]
Istiratumab (MM-141)	Advanced solid tumors	NCT01733004 phase I	Vomiting, nausea, fatigue, abdominal pain, increased AP, dyspnea, diarrhea, anemia, increased AST, and rash	Completed MM-141 was well tolerated as monotherapy and in combination with everolimus or paclitaxel + gemcitabine in patients with relapsed/refractory solid tumors	[180]
	Metastatic pancreatic cancer	NCT02399137 phase II	Neutropenia, alopecia, diarrhea, fatigue, thrombocytopenia, anemia, and decreased appetite	Completed Istiratumab failed to improve the efficacy of chemotherapy	[175]
	CRC, NSCLC, and HNSCC	NCT02538627 phase I		Terminated	
Duligotuzumab	Locally advanced or metastatic solid tumors with mutant KRAS	NCT01986166 phase I	Diarrhea, general disorders, dermatitis acneiform, rash, rash erythematous, rash maculo-papular, and nausea	Completed The combination of cobimetinib and duligotuzumab was correlated with increased toxicity and limited efficacy	[181]
	Locally advanced or metastatic epithelial tumors	NCT01207323 phase I	Headache, rash, and diarrhea	Completed Duligotuzumab was well tolerated with evidence of tumor pharmacodynamic modulation and antitumor activity in HNSCC	[182]
	Recurrent/metastatic HNSCC	NCT01911598 phase I	Neutropenia, hypokalemia, dehydration, anemia, and diarrhea in arm A and neutropenia, anemia, febrile neutropenia, leukopenia, thrombocytopenia, and hypomagnesemia in arm B	Completed Duligotuzumab with cisplatin + 5-fluorouracil (arm A) or carboplatin + paclitaxel (arm B) demonstrated promising activity despite chemotherapy dose reductions and could be maintained with duligotuzumab alone	[183]
	KRAS wild-type metastatic CRC	NCT01652482 phase II	Rash, diarrhea, fatigue, and nausea. There were fewer rash events of any grade in the duligotuzumab arm but more diarrhea	Completed The combination of FOLFIRI with duligotuzumab did not improve clinical outcomes compared with the cetuximab combination	[174]
	Recurrent/metastatic HNSCC	NCT01577173 phase II	Rash, infections, diarrhea, fatigue, and nausea	Completed Duligotuzumab demonstrated similar activity to cetuximab, but not superior	[173]

NSCLC: non-small cell lung cancer, BC: breast cancer, HNSCC: head and neck squamous cell carcinoma; AEs: adverse events; PFS: progression-free survival; ESCC: esophageal squamous cell carcinoma; CRC: colorectal cancer; OS: overall survival.

### 8.3. HER3-Targeting Antibody–Drug Conjugates and Clinical Trials

The new era of therapy is antibody–drug conjugates (ADCs), where cytotoxic small-molecule inhibitors conjugated on a monoclonal antibody scaffold; upon binding with the cell surface antigen, the ADC is internalized by the tumor cell and processed by the endo-lysosomal system. Patritumab deruxtecan (U3 1402; HER3-DXd) is one of the ADCs (Table 4), where the topoisomerase I inhibitor deruxtecan is attached with patritumab via a tetrapeptide-based cleavable linker. This highly cytotoxic patritumab deruxtecan is found to inhibit DNA replication and trigger immune cells. A study evaluated its antitumor activity against HER3-expressing tumors with tolerable safety profiles [184]. In a preclinical study, patritumab deruxtecan significantly sensitized the tumor to PD-1 blockade, which further suggests patritumab deruxtecan is a promising candidate as a partner of immunotherapy for patients with tumor-specific HER3 expression [185]. 

#### 8.3.1. Development of Antibody–Drug Conjugates in Breast Cancer

In a first-in-human study (U31402-A-J101), HER3-DXd was evaluated in heavily pretreated HER3-expressing metastatic breast cancer patients [191]. This study found promising antitumor activity in HR+/HER2− and HER2+ metastatic breast cancer patients as well as TNBC patients. HER3-DXd also showed a manageable toxicity profile and a low rate of discontinuation (9.9%) due to treatment-emergent adverse events (TEAEs), most commonly gastrointestinal and hematologic.

Based on the promising results of the first-in-human study (U31402-A-J101), HER3-DXd was investigated in HER2-positive breast cancer treated with chemo-free dual HER2 blockade. This study showed a combined score of TILs, and tumor cellularity (CelTIL score) was correlated with a pathological complete response at day 15 [192]. SOLTI TOT-HER3 window of opportunity trial (NCT04610528) showed a single dose of HER3-DXd (6.4 mg/kg) led to a clinically meaningful response, increased immune infiltration, and suppression of proliferation across varied levels of baseline ERBB3 mRNA [193]. The TOT-HER3 trial is still enrolling patients to evaluate the biological activity of HER3-DXd, measured as the CelTIL score increased post-treatment (C1D21) in patients with HR+/HER2-negative breast cancer and patients with TNBC tumors. Additionally, another phase II ICARUS-BREAST trial (NCT04965766) is investigating the HER3-DXd in hormone receptor-positive (HR+) unresectable locally advanced or metastatic breast cancer who are resistant to endocrine therapy and cyclin-dependent kinases 4 and 6 (CDK4/6) inhibitors. 

#### 8.3.2. Development of Antibody–Drug Conjugates in NSCLC

In a dose-expansion cohort (DEC) of a phase I trial (clinical trial information: NCT03260491), HER3-DXd exhibited an objective response rate (ORR) of 39% (95% CI 24.4–54.5%) in patients with *EGFR*-mutated NSCLC progressed after osimertinib and platinum-based chemotherapy [186]. In this study, HER3-DXd showed antitumor activity independent of EGFR TKI resistance mechanisms, suggesting that HER3-DXd could be considered a treatment option agnostic to the EGFR TKI resistance mechanism. After the progression of this study, another phase II trial, HERTHENA-Lung01 (clinical trial information: NCT04619004), is currently undergoing, which is investigating the effect of the HER3-DXd in previously treated patients with metastatic or locally advanced EGFR-mutated NSCLC after failure of third-generation TKIs. In a phase I trial (clinicalTrials.gov (accessed on 20 October 2023) Id # NCT04676477), HER3-DXd combined with osimertinib was also administered in patients with locally advanced or metastatic EGFR-mutated NSCLC. Interestingly, HER3-DXd was found to show effectiveness in many other oncogenic mutations, including *KRAS*/*NRAS* mutations and *ALK* fusions with limited TRAE and drug-related deaths [194].

## 9. Emerging Treatment Strategies

Many non-target strategies have been proposed to kill cancer cells [195,196]. Similarly, many strategies have been proposed that inhibit HER3 indirectly. Since some proteins such as HER3 are challenging to target, PROteolysis TArgeting Chimeras (PROTACs) has emerged as a possible solution that inhibits protein function by degrading target proteins instead of inhibiting them [197]. Although PROTAC-mediated HER3 degradation has not been reported, partial HER3 degradation was found via the treatment of monoclonal antibody NG33 [198]. Crosslinked trastuzumab binding to HER2 also caused HER2 and HER3 endocytosis and degradation in breast cancer cells [199]. Another study showed that treatment with TX2-121-1 covalently binds to HER3 and causes partial degradation of HER3, and inhibits heterodimerization of HER3 with HER2 or c-Met [200]. Different treatment strategies have emerged using noncoding RNAs [201,202,203]. Some studies also proposed antisense oligonucleotides or micro-RNA-mediated degradation of HER3 mRNA; however, these strategies have yet to be tested in clinical trials [204,205]. 

HER3-targeting vaccine (Ad-HER3-FL) was also reported to activate HER3-specific T-cells and induce anti-HER3-specific antibodies. This vaccine also enhanced intratumoral T-cell infiltration when combined with an immune checkpoint inhibitor compared to the vaccine alone [2]. Similarly, another study proposed antitumor immune responses via treatment with an immunoreacting HER3 epitope (HER-3872-868) in lung and head and neck cancer models [3]. 

## 10. Conclusions

The recent improvements in cancer research showed the importance of HER3 tumorigenesis, progression, and primary/acquired resistance to HER2- or EGFR-targeted therapy. Unlike other HER family receptors, inactive KD makes HER3 an obligate binding partner with other receptors. This feature gives researchers a challenging job in creating drugs against HER3. Many approaches have been proposed to inhibit the oncogenic function of HER3. First, due to the correlation of HER3 expression with sensitivity and resistance to HER TKIs, HER3 can be used as an important biomarker. Second, there is much progress in combination therapy with HER3 and EGFR/HER2-targeted agents, showing the way of overcoming the resistance. Thirdly, because HER3 signals through the PI3 K/Akt pathway, the combination therapy with HER3 and PI3 K/Akt inhibitors should have an anti-cancer function. Lastly, inhibition of HER3 function can also be achieved via inhibition of HER3-ligand (heregulin and neuregulin) production. 

## Figures and Tables

**Table 4:** Development of HER3-directed antibody–drug conjugates.

Antibody–Drug Conjugates	Study Population	Clinical Trial Phase	Adverse Events	Status Finding	Reference
U3-1402	Advanced or metastatic CRC	NCT04479436 phase II		Terminated	
	Naïve patients with HR + /HER2-early BC	NCT04610528 phase I		Active, not recruiting	
	Metastatic or unresectable NSCLC	NCT03260491 phase I	Nausea, vomiting, fatigue, decreased appetite, and alopecia	Active, not recruiting U3-1402 has antitumor activity and a manageable safety profile	[186,187,188]
	HER3 + metastatic BC	NCT02980341 phase I + II	Nausea, vomiting, and decreased appetite	Active, not recruiting In a preliminary analysis, U3-1402 demonstrated antitumor activity and a manageable safety profile	[189,190]
	Metastatic or locally advanced EGFR-mutated NSCLC	NCT04619004 phase II		Active, not recruiting	
	Locally advanced or metastatic EGFR-mutated NSCLC	NCT04676477 phase I		Recruiting	
	Metastatic BC	NCT04699630 phase II		Recruiting	
	Advanced BC	NCT04965766 phase II		Recruiting	
	Metastatic or locally advanced EGFR-mutated NSCLC after failure of EGFR TKI therapy	NCT05338970 phase III		Recruiting	

NSCLC: non-small cell lung cancer, BC: breast cancer, HNSCC: head and neck squamous cell carcinoma; AEs: adverse events; PFS: progression-free survival; ESCC: esophageal squamous cell carcinoma; CRC: colorectal cancer; OS: overall survival; TKI: tyrosine kinase inhibitor.

## Abbreviations

ADC	Antibody–drug conjugate
AE(s)	Adverse event(s)
bAbs	Bispecific antibodies
BC	Breast cancer
ECD	Extracellular domain
EGFR	Epidermal growth factor receptor
ER +	Estrogen receptor positive
ESCC	Esophageal squamous cell carcinoma
HER2 +	HER2-amplified
HNSCC	Head and neck squamous cell carcinoma
HRG	Heregulin
IGF1R	Insulin-like growth factor 1 receptor
IMA	Invasive mucinous adenocarcinomas
MoAbs	Monoclonals antibodies
NRG(s)	Neuregulin(s)
NSCLC	Non-small cell lung cancers
OS	Overall survival
PD-1	Programmed cell death-1
PFS	Progression-free survival
PR	Partial response
RAI	Radioactive iodine
RP2D	Recommended phase 2 dose
RTK	Receptor tyrosine kinases
scDb	Bispecific single-chain diabody
T-DM1	Trastuzumab-emtansine
TKIs	Tyrosine kinase inhibitors
TNBC	Triple-negative breast cancer
